# Developing Strain-Specific Simple Sequence Repeat (SSR) Markers for *Chlorella sorokiniana*

**DOI:** 10.4014/jmb.2404.04024

**Published:** 2024-07-18

**Authors:** Mais Sweiss, Maen Hasan, Nidal Odat

**Affiliations:** 1 Department of Biotechnology, Faculty of Agricultural Technology, Al-Balqa Applied University, Al-Salt 19117, Jordan; 2 Department of Plant Production and Protection, Faculty of Agricultural Technology, Al-Balqa Applied University, Al-Salt 19117, Jordan; 3 Department Medical Laboratory Sciences, Faculty of Science, Al-Balqa Applied University, Al-Salt 19117, Jordan

**Keywords:** *Chlorella*, simple sequence repeats, microsatellites, strain-specific

## Abstract

*Chlorella sorokiniana* green microalga offers many environmentally friendly applications, including wastewater treatment, biofertilizers, animal feed, and biofuel production. Different strains of *C. sorokiniana* have unique properties that may suit one application but not another. There is a need to distinguish between the many available strains of *C. sorokiniana* to choose the one that best fits the application. Consequently, our research goal was to develop strain-specific simple sequence repeat (SSR) markers to differentiate between the different strains. Seventeen markers spanning ten out of the twelve chromosomes of the *C. sorokiniana* genome were developed and validated on eight different strains from culture collections and our lab, and were then analyzed by fragment analysis. The results demonstrate the potential of these polymorphic markers to detect the genetic differences between the strains of *C. sorokiniana*, and to serve as useful tools for the intra-species population genetic analysis and conservation genetics studies of *C. sorokiniana*.

## Introduction

*Chlorella*, a widely known genus of microalgae primarily found in freshwater, but with some species inhabiting marine environments, soils, or living as endosymbionts [1], presents promising applications in various fields, such as biofuel production, due to its high growth rate and lipid content [2]. Some *Chlorella* species proved to be efficient in removing nutrients and metals from wastewater [3, 4], while others can be used as food supplements and animal feed [5, 6].

However, identifying *Chlorella* members at the species level based on the morphology is challenging due to limited morphological distinctions [1], exacerbated by environmental influences on morphology [7, 8]. For these reasons, polyphasic identification methods integrating morphological, ultrastructural, and molecular approaches are recommended [9, 10]. Since identifying *Chlorella* members at the species level is hard, it is even harder to identify different strains of the algae.

To address this challenge, microsatellite markers, a subgroup of tandem repeats in the genome known for their high mutation rates [11, 12], offer a solution for strain differentiation as they are very effective for selecting a target organism from its closely related species [13]. The discrimination power of the simple sequence repeat (SSR) markers depends on their polymorphic distribution in specific loci [13-15]. The specific flanking regions close to the microsatellite can provide accurate genotyping of intra- or inter-specific hybrids with a primer set [13, 16].

Selection of highly productive algal strains with favorable characteristics is a very important step to establish a successful, and economically viable, large-scale cultivation system. Understanding strain-specific differences in genome content and architecture is a significant issue in this field [17]. Microsatellite markers are a powerful tool for intraspecies level identification [13]. However, no microsatellite markers have been developed for *C. sorokiniana* strains yet. Thus, we sought in this research to develop SSR microsatellite markers to differentiate between *C. sorokiniana* strains.

## Materials and Methods

### *Chlorella* Strains

A total of nine *Chlorella* strains were used in this research. Three strains of *C. sorokiniana*, UTEX B 3016, UTEX 3010, and UTEX B 2805, were obtained from the culture collection of the University of Texas (UTEX) in the USA. Another two strains, *C. sorokiniana* SAG 211-31 and SAG 211-32, were obtained from the culture collection of algae at Goettingen University (SAG) in Germany. One strain of *C. sorokiniana*, NIES-2169, was obtained from the microbial culture collection of the National Institute for Environmental Studies (NIES) in Japan and used as the positive control. Strain NIES-2169 is the same as strain UTEX 1230, which its genome was used to develop SSR markers. In addition, two strains of *C. sorokiniana* were available in our lab, Somerton-3, isolated from the United Kingdom, and Jordan-40, isolated from Jordan [18]. The strain *C. vulgaris* CCALA 269, used as a negative control, was obtained from the culture collection of autotrophic organisms in the Czech Republic. All the strains were cultured on Bold’s Basal Medium (BBM) plates with 1% agar [19], and grown at 22°C with a light:dark cycle of 18:6 h at a light intensity of 60-80 μmol photon/m^2^/s.

### DNA Isolation

A single colony from each strain was cultured in 10 mL BBM under the following conditions: light:dark cycle of 16:8 h, light intensity of 120-150 μmol photon/m^2^/s, 22°C, and mixing at 250 rpm. After the culture bloomed, the DNA was isolated following the protocol of Doyle and Doyle (1987) with minor modifications [20]. Around 30 × 10^6^ cells were collected and homogenized in liquid nitrogen. Then, 750 μl of preheated (60°C) CTAB isolation buffer [2% hexadecyltrimethylammonium bromide (CTAB), 20 mM Ethylenediaminetetraacetic acid (EDTA), 1.4 M NaCl, 100 mM Tris-HCl pH 8.0] was used to lyse the cells. The samples were then incubated at 60°C for 30 min with gentle mixing, followed by purification with the same volume of chloroform, and centrifugation at 8,000 ×*g* for 10 min. Next, the nucleic acids were precipitated by adding the same volume of ice-cold isopropanol, followed by incubation at -20°C for 30 min, and then collection by centrifugation for 15 min at 15,000 ×*g*. The pellet was washed with 500 μl of 80% ethanol and dried, and then it was resuspended in 40 μl of 1X TE buffer (10 mM Tris-HCl, 1 mM EDTA, pH 8.0). Subsequently, the isolated genomic DNA was analyzed on 0.7% agarose gel in 0.5X TBE running buffer, stained with 0.5 μg/ml ethidium bromide.

### Amplification of Internal Transcribed Spacer (ITS) Region and the Plastid Elongation Factor Tu (*tufA*) Gene

To amplify the ITS1-5.8S-ITS2 region, the ITS-F 5`-GAAGTCGTAACAAGGTTTCC-3` and ITS-R 5`-TCCTGGTTAGTTTCTTTTCC-3` primers were used [21]. Whilst, for the chloroplast *tufA* gene amplification, tufAF 5`-TGAAACAGAAMAWCGTCATTATGC-3` and tufAR 5`-CCTTCNCGAATMGCRAAWCGC-3` primers were used [22]. The PCR mixes were prepared according to the instructions of the Phusion High-Fidelity DNA Polymerase Kit (New England BioLabs Inc., USA). In a total volume of 25 μl containing 50-250 ng genomic DNA, dNTPs 240 μM, from each primer 0.4 μM; 1X of 5X Phusion GC Buffer, 3% DMSO, and 0.5 U of the high-fidelity DNA polymerase. The thermal cycling conditions were initial denaturation at 94°C for 4 min, followed by 30 cycles of denaturation at 94°C for 45 s, annealing at 57°C for the ITS region, and 58°C for *tufA* gene for 45 s. The extension step was at 72°C for 30 s, followed by a final extension at 72°C for 7 min using Prime Thermal Cycler (Prime, UK). The results were analyzed on 1% agarose gel stained with 0.5 μg/ml ethidium bromide.

### Sequencing

The PCR products were purified using an agarose gel extraction kit from Jena Bioscience (Jena Bioscience, Germany), and sent for sequencing by Macrogen (Macrogen, Republic of Korea). For each PCR product, two sequences were obtained, one for the forward and one for the reverse reaction. After that, the low-quality sequences were removed by trimming these sequences from both the beginning and the end, and then the two sequences, the forward and the reverse complement of the reverse, were aligned to obtain the reliable sequence for each PCR product.

### Phylogenetic Analysis based on the ITS Region and *tufA* Gene

The identity of the obtained strains was confirmed by doing a search using the Nucleotide Basic Local Alignment Search Tool (Nucleotide BLAST), provided by the National Center for Biotechnology Information (NCBI) [23, 24]. To elucidate the phylogenetic relationships between the *Chlorella* strains based on the two amplified regions, a maximum likelihood method based on the Tamura-Nei model was used to construct the phylogenetic trees using MEGA 11 [25, 26].

### Searching for SSRs and Designing Strain-Specific Primers

The whole-genome sequence of *C. sorokiniana* strain UTEX 1230 (NIES-2169) was obtained from the National Center for Biotechnology Information (NCBI) database (http://www.ncbi.nlm.nih.gov/) under the GenBank assembly Accession No. GCA_003130725.1. The candidates for the microsatellite motifs were screened using the Unipro UGENE program [27]. Strain-specific primers were designed based on the upstream/downstream flanking sequences of the chosen microsatellite motifs using Primer3Plus [28]. The criteria for primer design included the prediction of annealing temperature (*Tm*) of 55°C-61°C, primer length ranging between 18-27 bp, PCR amplicon lengths of 200-1000 bp when possible, and an annealing temperature difference between both pairs of primers of under 1°C. Primers that are self-complementary and complementary to each other were not chosen. For fragment analysis, on the 5` end of the forward primers, the M13 complementary sequence was added to allow the attachment of the M13 6 FAM-labeled primer.

### SSR PCR Amplification and Genotyping

The seventeen pairs of the SSR primers covered most of the chromosomes, except chromosomes 4 and 12, and were verified on eight strains of *C. sorokiniana* using touchdown PCR. The PCR reactions were performed in a Prime Thermal Cycler (Prime) in a total volume of 25 μl using AmpliTaq Gold 360 Master Mix Kit (Applied Biosystems, USA) according to the manufacturer’s instructions containing 20-50 ng of genomic DNA, 1X AmpliTaq Gold 360 Master Mix, 1 μl of 360 GC Enhancer, 0.2 μM of reverse primer, and 0.2 μM of the forward primer mixture (the primers forward 1:3 of the volume and an M13 6 FAM-labeled primer for the rest of the volume).

Touchdown PCR was applied to increase the specificity and sensitivity of the reaction, in which the initial denaturation step was at 95°C for 4 min. Stage 1 consisted of 5 cycles of denaturation at 95°C for 45 s, with annealing starting at 68°C for 2 min with decreases of 2°C per cycle, and an extension step at 72°C for 1 min. Stage 2 consisted also of 5 cycles of denaturation at 95°C for 45 s, with annealing starting at 58°C for 1 min with decreases of 2°C per cycle, and an extension step at 72°C for 1 min. The third stage consisted of 27 cycles of denaturation at 95°C for 45 s, annealing at 52°C for 1 min, an extension step at 72°C for 1 min, and a final extension step at 72°C for 10 min. The quality and quantity of PCR products were validated on a 1.2% agarose gel stained with 0.5 μg/ml ethidium bromide.

### Fragment Analysis

To detect the precise size of the amplified PCR products, the PCR products were sent for fragment analysis by Macrogen (Macrogen) to be analyzed by 3730xl DNA (ABI, USA) Analyzer. The results were analyzed using Peak Scanner v1.0 (Applied Biosystems).

### Data Analysis

Polymorphic amplified SSR markers were classified as either present (1) or absent (0), and then transformed into a binary data matrix. To detect the relationship between the eight studied strains of *C. sorokiniana*, genetic similarity was estimated following the Dice similarity coefficient [29], which was calculated between all possible pairs of strains by using the SimQual method implanted in NTSYSpc (Version 2.01, Exeter Software, Setauket, NY, USA). Based on this similarity matrix, a tree showing the relationships among the different *C. sorokiniana* strains was constructed by the unweighted pair group method with arithmetic average (UPGMA) using the SAHN modules in the same software.

## Results

### Phylogenetic Analysis Based on the ITS Region and *tufA* Gene

To confirm the identity of the obtained C. Sorokiniana strains, two DNA barcoding markers were chosen, the ITS region and *tufA* gene, which were amplified and sent for sequencing. The ITS region was selected because its nuclear rDNA substitution rates are much higher than those of the rRNA genes, and it was also recommended as a marker for species identification of macro- and microalgae. In addition, *tufA* was chosen because it is recommended as a marker for DNA-based species delimitation and/or barcoding in green algae [30].

**Fig. 1:**
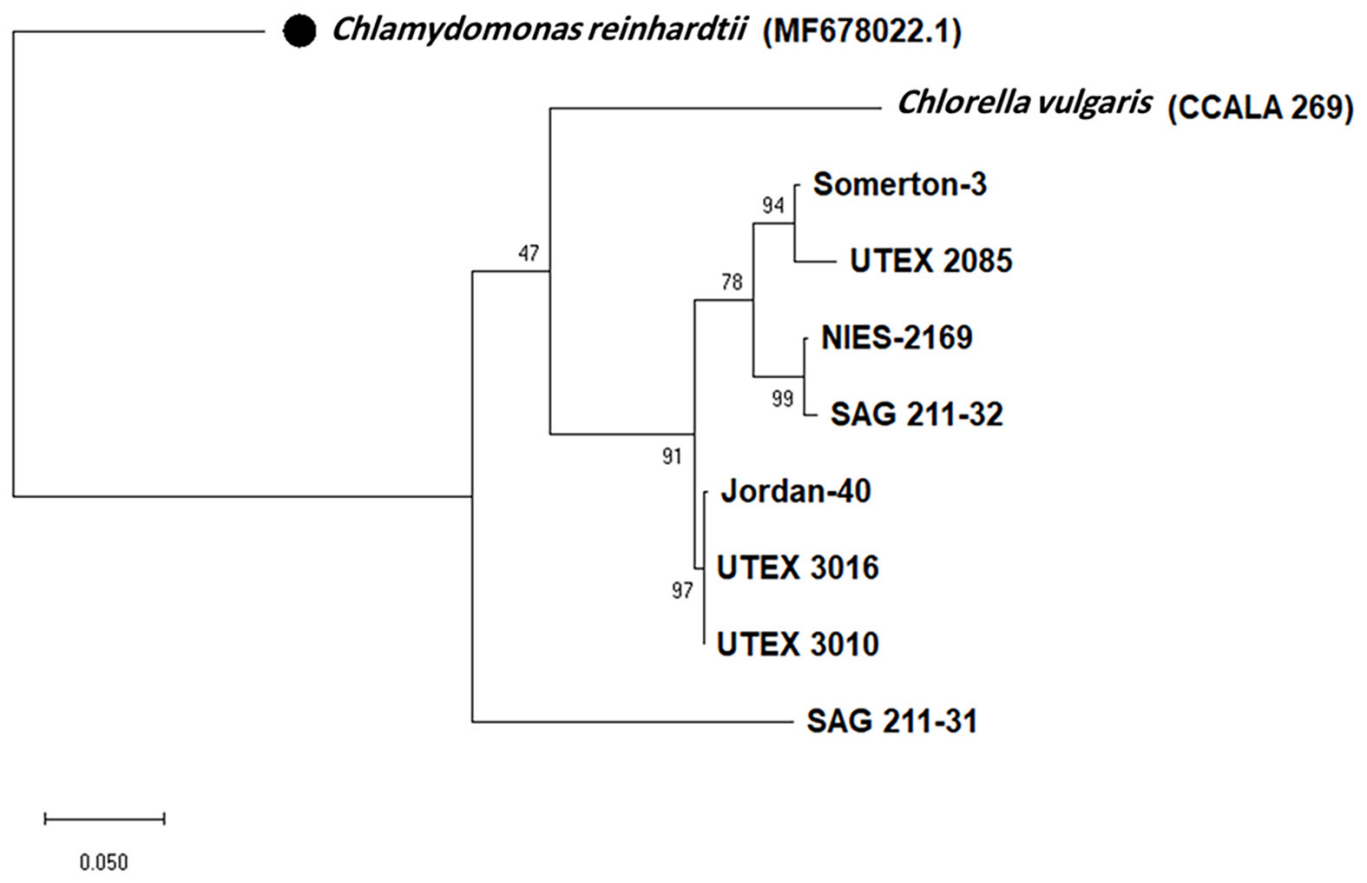
Maximum likelihood phylogenetic tree of ITS region. The tree was constructed using MEGA11, the bootstrap value was 10000, and the black circle is for outgroup used to root the tree, which is *Chlamydomonas reinhardtii* (Accession No. MF678022.1).

**Fig. 2:**
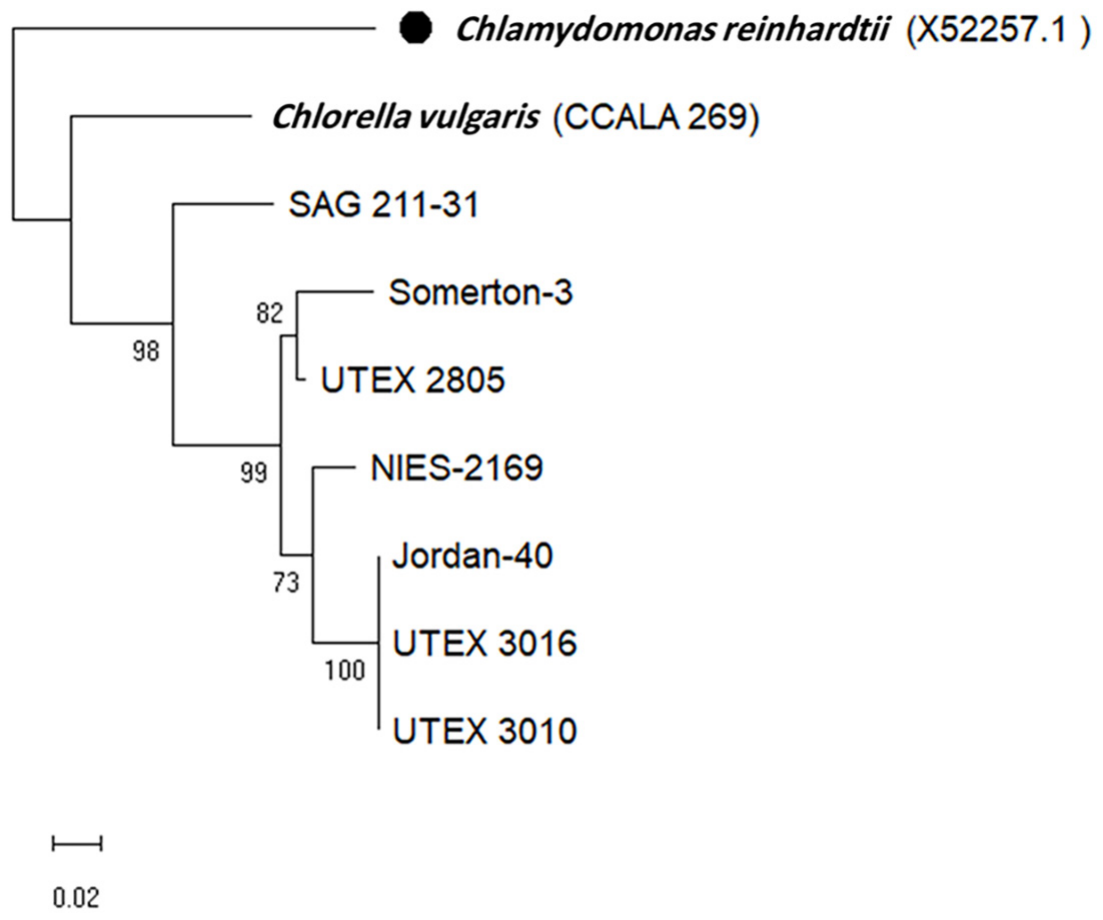
Maximum likelihood phylogenetic tree of *tufA* gene. The tree was constructed using MEGA11, the bootstrap value was 10000, and the black circle is for the outgroup used to root the tree which is *Chlamydomonas reinhardtii* (Accession No. X52257.1).

The identification of the *C. sorokiniana* strains based only on the BLAST search for their ITS and *tufA* sequencing was challenging, because of the limited sequences available in the database. The phylogenetic analysis for the obtained sequences for the ITS region and *tufA* gene can show differences at the strain levels, and the phylogenetic tree was rooted in the outgroup *Chlamydomonas reinhardtii* sequences which were obtained from GenBank. Some strains are closely related to each other and cluster together, such as UTEX 3010, UTEX 3016, and Jordan-40, as well as Somerton-3 and UTEX 2805 (Figs. 1 and 2), while SAG 211-31 stands alone on separate branches indicating that it is different from the rest of the strains based on the two loci. We remind the reader that the aim of these trees was not to study the genetic variation, but only to confirm the identity of the strains before we began the SSR marker validation.

### Validation of SSR Primer Design

**Fig. 3:**
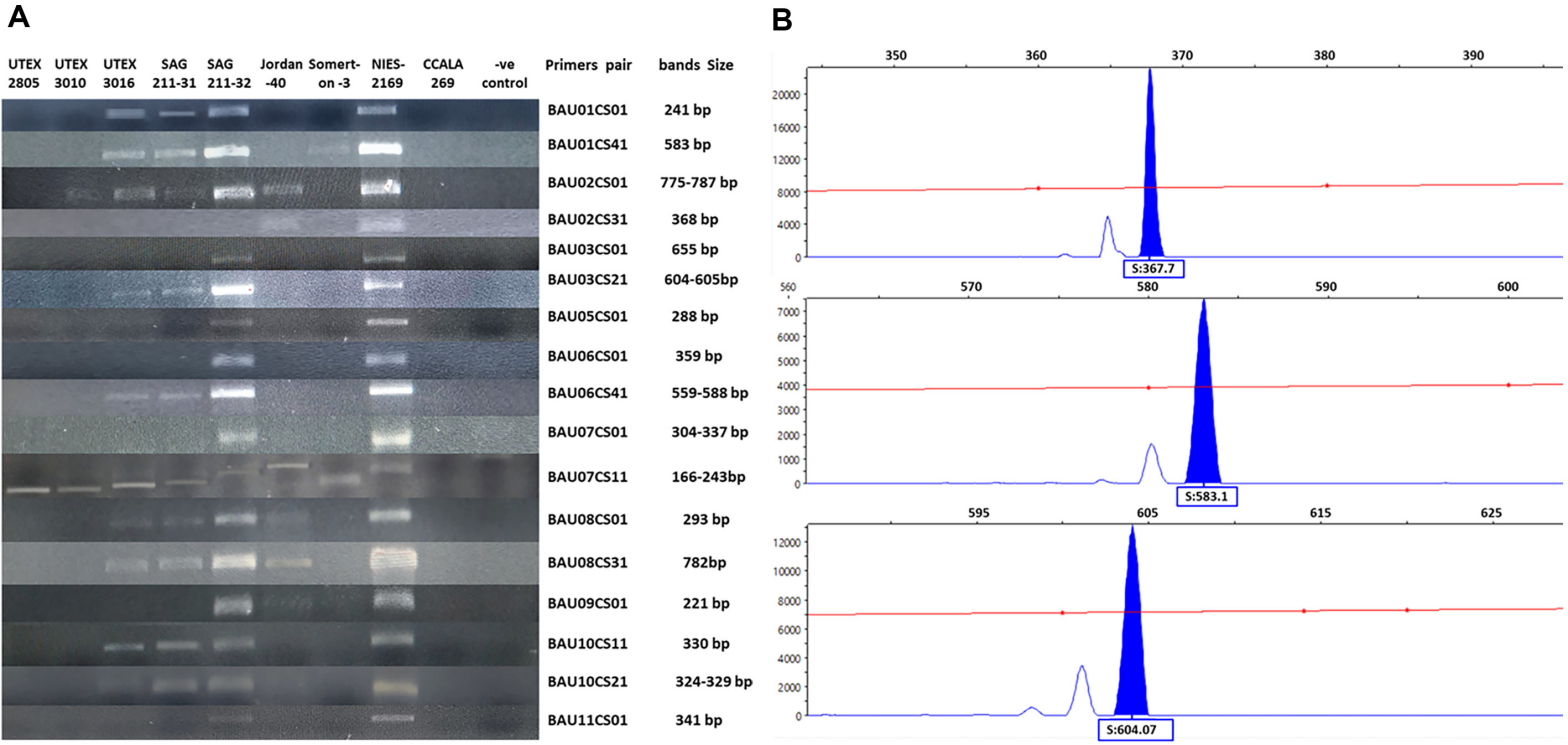
SSR primer validation and fragment analysis. (**A**) Touchdown PCR results were analyzed on 1.2% agarose gel stained with 0.5μg/ml ethidium bromide, NIES-2169, *C. sorokiniana* strain positive control; CCALA 269, *C. vulgaris* a negative control; -ve control, master mix without DNA. (**B**) Fragment analysis results for some samples, band size is shown in a box below the peak.

**Fig. 4:**
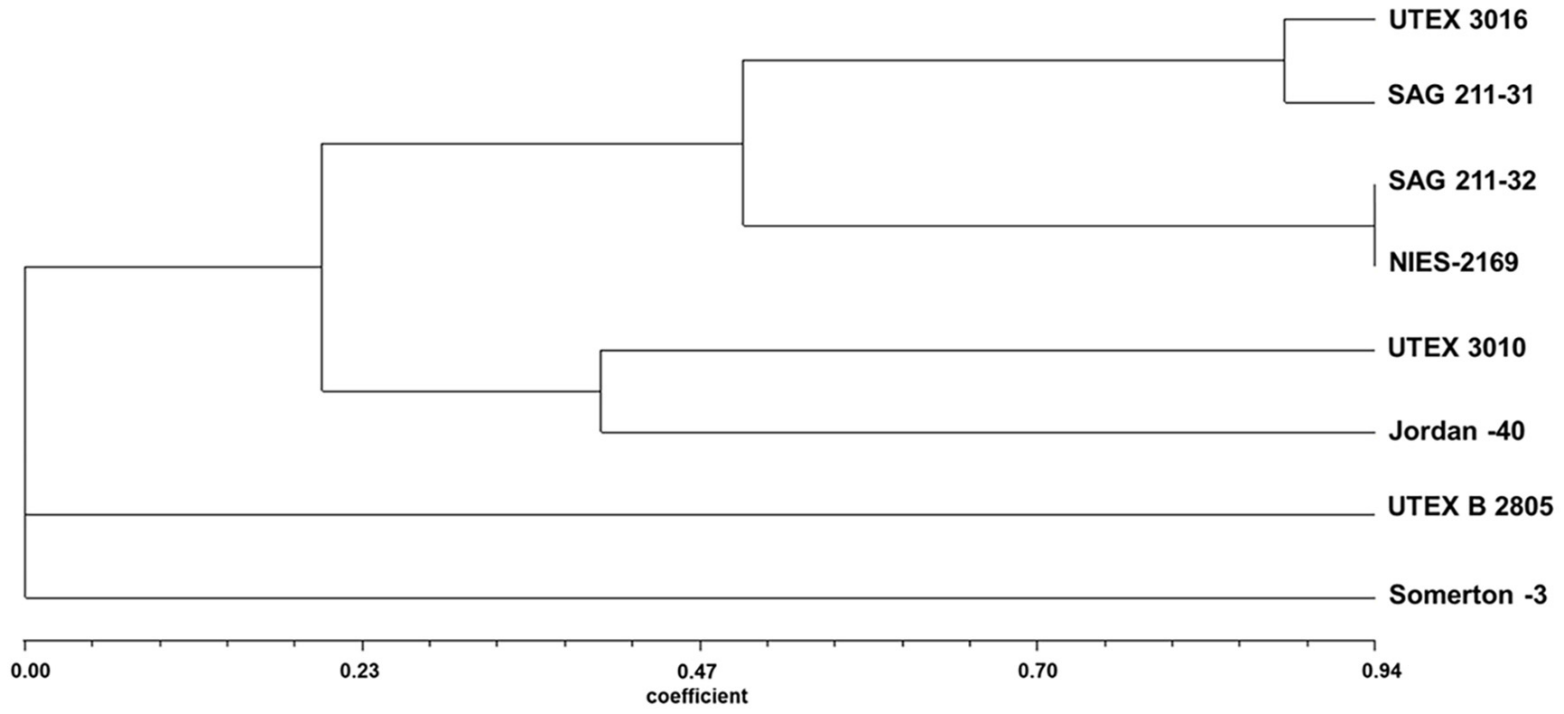
UPGMA Tree of eight *C. sorokiniana* strains based on UPGMA analysis of SSR markers. The SSR markers represent 17 different loci of the whole genome. The tree was constructed using NTSYSpc software.

Seventeen pairs of primers that present in ten out of the twelve chromosomes of *C. sorokiniana* genome were designed and tested. These primers were validated on seven different *C. sorokiniana* strains and the positive control (NIES-2169), the genome sequence of which was used to design the primers, so it should show positive results, in addition to a negative control of a different *Chlorella* strain (CCALA 269) that should be negative (Fig. 3A). After the touchdown PCR, the PCR products were subjected to fragment analysis to detect the precise size of the amplified fragment (Fig. 3B). The results are summarized in Table 1. All these markers showed polymorphism among the eight examined strains (Table 1). Genetic similarity was assessed by the Dice similarity coefficient, and based on the similarity results, the Dice genetic similarity for all investigated strains ranged from 0.000 to 0.9375, with an overall mean of 0.191 (Table 2). The UPGMA tree represents the genetic similarity of the eight examined strains on 17 different loci distributed amongst the whole genome, and it showed that UTEX 2805 and Somerton-3 stand alone in different branches, indicating that they have the lowest similarity with the other strains based on the developed SSR markers (Fig. 4). On the other hand, SAG 211-32 and NIES-2169 have the highest genetic similarity observed (Table 2). Furthermore, SAG 211-31 is more similar to UTEX 3016 based on the developed SSR marker (Fig. 4).

## Discussion

Eight different strains of *C. sorokiniana* were studied in this research. Six of these were obtained from culture collections while two came from our lab, and were isolated from wastewater from Jordan and the UK. Before starting the experiment, the strains were subjected to DNA barcoding and BLAST search to ensure that they were *C. sorokiniana*, and that there was no contamination or other problems. A phylogenetic tree for each DNA barcode was then constructed to confirm the identity results. These results represent the phylogenetic relationship among the isolated strains using one locus only (either ITS or *tufA*). To study the differences among the whole genome, 17 SSR markers were developed. SSR markers are a very valuable tool in studying the evolutionary process, population structure, and genetic mapping [11]. These primers were polymorphic and were able to show the genetic diversity between *C. sorokiniana* strains. The low similarity among some strains of *C. sorokiniana* in Fig. 4 has been reported by a previous comparative study on the genome of three *C. sorokiniana* strains, UTEX 1230 (NIES-2169), 1228, and UTEX 3016. The study found that less than 15% of the genome has nucleotide identity of 80%, which raises questions on the species taxonomy of the *C. sorokiniana* strains [17].

*C. sorokiniana* is a non-motile, single-cell organism that is directly in contact with the environment, thus the selection pressure is high among the cells. Moreover, *Chlorella* members are small in size, ranging from 2 to 15 μm in diameter; they reproduce asexually within a few hours and can form large populations in a short period [31]. Besides that, they are haploid [32], and consequently, spontaneous mutations can be detected and traced within a few months in the cultured microalgae [33]. The genome sequence of *C. sorokiniana* strains revealed the presence of around 71 sex-related and meiosis-related genes, and a further 25 homologous recombination genes were found, which suggests that *C. sorokiniana* may be capable of dividing by meiosis and engage in homologous recombination [17]. In addition, genome sequencing discovered the presence of hotspots for genomic rearrangements and inversion in their genome [34]. The acquisition of DNA sequences from other organisms such as viruses has been documented in algae. More than 90,000 viral-origin sequences in 184 algal genomes have been detected, and thus viruses have played a role in the adaptation of their host to different environments [35]. These reasons and others have contributed to the high genetic diversity of *Chlorella* and allows them to adapt easily to selection pressure and environmental changes [33].

The increase of genetic diversity in a population is advantageous since it increases tolerance to harsh environmental conditions, and it is an important factor for maintaining ecological functions under varying environmental conditions [36]. This genetic diversity may explain the ability of different strains of *C. sorokiniana*, such as thermotolerance strains, to tolerate different environmental conditions. In addition to its thermotolerance, a *C. sorokiniana* strain that was isolated from water bodies around a steel plant in India can also tolerate high levels of carbon dioxide and nitric oxide [37]. Other thermotolerant strains, like *C. sorokiniana* LWG002615, which was isolated from Jeori thermal spring in India [38], and UTEX 2085, were found to grow at temperatures of 40–42°C and tolerate high light intensity of 2,500 μmol photon/m^2^/s for 5 h daily [39, 40]. Furthermore, some strains of *C. sorokiniana* can tolerate relatively high CO_2_ concentrations [41, 42], and grow in wastewater. Three strains of *C. sorokiniana*, CS-01, UTEX 2714, and UTEX 1230(NIES-2169), behaved in different ways when they were grown in 10% diluted effluent of anaerobic digester from cattle manure [43]. The genetic diversity also enables some strains of *C. sorokiniana* to accumulate large amounts of lipid. In a project looking for top-performing green microalgae strains that would be suitable for biofuel feedstocks and performed by the National Alliance for Advanced Biofuels and Bioproducts (NAABB) in the USA, *C. sorokiniana* strain DOE1412 (UTEX 3016) was a promising algal species for biofuel applications [2].

Several microalgal microsatellite markers have already been developed to study the genetic variation among the strains (Table 3) of some beneficial green microalgae. For instance, strain-specific SSR markers were developed for *C. vulgaris* and *C. pyrenoidosa* [13, 44]. Meanwhile, in case of *Haematococcus pluvialis*, the main astaxanthin-producing organism from different geographical regions, the strains were grouped based on their geographical locations [45]. In addition, seven microsatellite markers were identified and would be valuable for studying the genetic structure of the red alga *Porphyra umbilicalis* population [46]. On the other hand, microsatellites were used to study the genetic diversity of economically important harmful algae that cause economic loss, such as *Ulva (Enteromorpha) prolifera*, which causes green tides [47], or the toxic dinoflagellate *Alexandrium tamarense*, a cause of mass fish death in red tides [48, 49], and the dinoflagellate *Prorocentrum donghaiense*, whose blooms threaten coastal ecosystems [50]. Microsatellites were also applied to study the polymorphism of the different phases and sexes of the red alga *Gracilaria lemaneiformis* [51], and for species delimitation of *Cladophoraceae*, a species that is endemic to Lake Baikal [52]. A cost-effective microsatellite marker development method using Double-Digest Restriction site Associated DNA (ddRAD) was evaluated for six non-model species of red and brown seaweeds that have economic and ecological importance [53]. The microsatellite markers can be applied to study the chloroplast genome polymorphisms which is important for evolutionary studies. The presence of large numbers of SSRs may stimulate genome expansion, and a total of 401 SSRs were identified in the chloroplast genome of *H. pluvialis* and were dominated by mononucleotide repeats and tetranucleotide repeats [54].

## Conclusion

The genotyping of the eight *C. sorokiniana* strains based on the 17 developed SSR markers present in ten out of the twelve chromosomes of the genome suggests that while some strains are very closely related, such as SAG 211-32 and NIES-2169, there are two strains, Somerton-3 and UTEX 2805, that showed low similarity to the rest of the studied strains. This high genetic diversity among the strains may give these strains their unique characteristics, such as thermal tolerance. The developed SSR markers were able to successfully differentiate between the studied strains, and they could also provide a low-cost technique to further investigate the genetic diversity among the different *C. sorokiniana* strains.

## Figures and Tables

**Table 1:** The designed SSR primers and their results after fragment analysis without the negative controls.

	Primer name	Primer	Locus	Repeat motif	Size range (bp)	No. of non-amplifying samples
1	BAU01CS01_F BAU01CS01_R	GTAAAACGACGGCCAGCTCT TCAACATCTGCCATCACAAT AGGCGATGCTGGGTCTATG	Chromosome 1	(TGC)16	241	4
2	BAU01CS41_F BAU01CS41_R	GTAAAACGACGGCCAGAGC AGGGAGGAGGACGAGGACA GGGCAGTGACATAGGG	Chromosome 1	(GCA)15	583	3
3	BAU02CS01_F BAU02CS01_R	GTAAAACGACGGCCAGGGG ATGGTTGAGGGTGCTAGGAT GGCTGCTCTGCTC	Chromosome 2	(GCT)115	775-787	2
4	BAU02CS31_F BAU02CS31_R	GTAAAACGACGGCCAGCCAG CCGGTCAATCAGATCAGGGG ACAAGAGCAAGGC	Chromosome 2	(TGC)10	368	6
5	BAU03CS01_F BAU03CS01_R	GTAAAACGACGGCCAGCATA CTGGGAGCGGTGCGGTCCTC ACAGCAGCCTCAG	Chromosome 3	(GGC)5(GCT) 25	655	6
6	BAU03CS21_F BAU03CS21_R	GTAAAACGACGGCCAGGTG GATTGCTGCTGAGACGTGCT GGTATCCTGTGTAGCC	Chromosome 3	(TGC)20	604-605	4
7	BAU05CS01_F BAU05CS01_R	GTAAAACGACGGCCAGAAAC GACATCCAAAAGACCAACAA GACAGGAAGCACGATGGA	Chromosome 5	(TG)23	288	6
8	BAU06CS01_F BAU06CS01_R	GTAAAACGACGGCCAGAAA AGTCGCCAGAAATCCACCTG CTGGAACTGCTGAGGT	Chromosome 6	(GCA)21	359	6
9	BAU06CS41_F BAU06CS41_R	GTAAAACGACGGCCAGCTGT CAAAGCATCCCCATCGTGTT TGCTATTTGGGCGGAC	Chromosome 6	(CAG)22	559-588	4
10	BAU07CS01_F BAU07CS01_R	GTAAAACGACGGCCAGGCTG GACTTGATGCCCTGAGTGTA CTGGCTGGCGATCT	Chromosome 7	(GCT)48	304-337	6
11	BAU07CS11_F BAU07CS11_R	GTAAAACGACGGCCAGCCG ACAAATCTAACCGCCCTCAG CAGCAGACGACCAGC	Chromosome 7	(CTG)24	166-243	0
12	BAU08CS01_F BAU08CS01_R	GTAAAACGACGGCCAGCTGT GGTCGGCAGGATTGAAATGA CGGGAGTGAGAATGG	Chromosome 8	(CTG)28	293	3
13	BAU08CS31_F BAU08CS31_R	GTAAAACGACGGCCAGGCCT CTCCACCTCCACACAGCGAA ACCACGACAACAG	Chromosome 8	(TGC)25	782	3
14	BAU09CS01_F BAU09CS01_R	GTAAAACGACGGCCAGGCAC GACTTGATCTTGGCAACCTT GGCGGTGGTTAATTGT	Chromosome 9	(TCT)19AC (TCT)4	221	5
15	BAU10CS11_F BAU10CS11_R	GTAAAACGACGGCCAGACCA ACCGTCACACTTTCTCGAGC GAGGATTGAAGCAG	Chromosome 10	(CA)32	330	4
16	BAU10CS21_F BAU10CS21_R	GTAAAACGACGGCCAGGCG AGATGGGGTCAGGAGCTTAT TAGGGGCTGGGCAGA	Chromosome 10	(TG)55	324-329	4
17	BAU11CS01_F BAU11CS01_R	GTAAAACGACGGCCAGGCCT CGTGACCCAAACACCCCAGC ACGACTTTCATACC	Chromosome 11	(TG)29	341	6

**Table 2:** Dice similarity matrix among the analyzed 8 genotypes based on SSR molecular data.

	UTEX B 3016	UTEX 3010	UTEX 2805	SAG 211-31	SAG 211-32	Jordan -40	Somerton -3	NIES-2169
UTEX B 3016	1.000000							
UTEX 3010	0.222222	1.000000						
UTEX 2805	0.000000	0.000000	1.000000					
SAG 211-31	0.875000	0.000000	0.000000	1.000000				
SAG 211-32	0.521739	0.000000	0.000000	0.518518	1.000000			
Jordan -40	0.500000	0.400000	0.000000	0.307692	0.300000	1.000000		
Somerton -3	0.000000	0.000000	0.000000	0.000000	0.000000	0.000000	1.000000	
NIES-2169	0.476190	0.000000	0.000000	0.480000	0.937500	0.315789	0.000000	1.000000

**Table 3:** Summary of some of the microsatellites used to study genetic diversity in algae from literature listed in order from the oldest to the newest.

Algae	Microsatellite marker	Main feature	Year/Citation
*Gracilaria lemaneiformis*, *Gracilaria tenuistipitata* var. *liui*, *Gracilaria vermiculophylla*, *Gracilaria blodgettii* (Red macroalga)	Inter Simple Sequence Repeats (ISSR)^†^	Developed ISSR markers to investigate the genetic diversity of the genus *Gracilaria* (Rhodophyceae)	2003/ [55]
*Alexandrium tamarense* (Dinoflagellate microalgae)	Microsatellite *	Developed polymorphic microsatellite loci that provided microsatellite markers with high polymorphism	2004/ [49]
*Gracilaria lemaneiformis* (Red macroalga)	ISSR^†^	Used ISSR to distinguish and study the polymorphisms of the phases and sexes of *G. lemaneiformis*	2006/ [51]
*Alexandrium tamarense* (Dinoflagellate microalga)	Microsatellite*	Performed genetic analysis of *Alexandrium tamarense* populations from 10 sites along the Japanese and Korean coasts and also tried to detect the impact of natural and human-assisted dispersals on the genetic structure and gene flow	2007/ [48]
*C. vulgaris*, *C. pyrenoidosa* (Green microalgae)	ISSR	Used ISSR primers that were designed for *Triticum aestivum* to study genetic polymorphism and diversity of *Chlorella* for intra-species genetic analysis.	2008/ [44]
*Chondrus crispus* (Red macroalga)	ISSR^#^	ISSR analysis was used to investigate genetic variations of haploid and diploid samples from nine North Atlantic	2008/ [56]
*Haematococcus pluvialis* (Green microalga)	ISSR	Using ISSR and Random Amplified Polymorphic DNA (RAPD) markers to study genetic diversity	2011/ [45]
*Ulva (Enteromorpha) prolifera* (Green macroalga)	ISSR^#^	Using ISSR markers to provide information on the genetic variation of both attached and floating *U. prolifera* samples from China	2011/ [47]
*C. vulgaris* (Green microalga)	SSR	Developed and evaluated microsatellite markers along the chloroplast genome for strain-specific identification method	2014/ [13]
*Porphyra umbilicalis* (Red macroalga)	Microsatellite	Developed polymorphic microsatellite markers from enriched DNA libraries	2018/ [46]
*Prorocentrum donghaiense* (Dinoflagellate microalga)	SSR	Development of SSR marker based on transcriptome sequencing to study genetic diversity of the dinoflagellate blooms	2020/ [50]
Cladophoraceae species (Green macroalgae)	SSRs	Designed a set of SSRs for species delimitation and insights into ploidy species of Cladophoraceae using high-throughput sequencing (HTS) data of three morphospecies	2020/ [52]
*Haematococcus pluvialis* (Green microalga)	Repeats and SSR	Identified among the chloroplast genome, potentially useful markers for detecting polymorphism used in evolutionary studies, which has high-degree variations within the same species	2021/ [54]
*Alaria esculenta*, *Pylaiella littoralis* (Brown macroalgae) *Calliblepharis jubata*, *Gracilaria gracilis*, *Gracilaria dura*, *Palmaria palmata* (Red macroalgae)	Microsatellites	Identified and characterised microsatellites in genomic sequences obtained using Double-Digest Restriction site Associated DNA (ddRAD), provided preliminary data about the genetic structure and reproduction mode of these six nonmodel species	2023/ [53]

*Research used the same microsatellite marker

†Research used the same microsatellite marker

^#^Research used the same microsatellite marke
